# Short-Wavelength Light Sensitivity of Circadian, Pupillary, and Visual Awareness in Humans Lacking an Outer Retina

**DOI:** 10.1016/j.cub.2007.11.034

**Published:** 2007-12-18

**Authors:** Farhan H. Zaidi, Joseph T. Hull, Stuart N. Peirson, Katharina Wulff, Daniel Aeschbach, Joshua J. Gooley, George C. Brainard, Kevin Gregory-Evans, Joseph F. Rizzo, Charles A. Czeisler, Russell G. Foster, Merrick J. Moseley, Steven W. Lockley

**Affiliations:** 1 Division of Neuroscience and Mental Health, Faculty of Medicine, Imperial College London, London W6 8RF, United Kingdom; 2 Division of Sleep Medicine, Brigham and Women's Hospital, Boston, Massachusetts 02115; 3 Nuffield Laboratory of Ophthalmology, University of Oxford, Wellcome Trust Centre for Human Genetics, Roosevelt Drive, Oxford, OX3 7BN, United Kingdom; 4 Division of Sleep Medicine, Harvard Medical School, Boston, Massachusetts 02115; 5 Department of Neurology, Thomas Jefferson University, Philadelphia, Pennsylvania 19107; 6 Department of Ophthalmology, Massachusetts Eye and Ear Infirmary, Harvard Medical School, Boston, Massachusetts 02114; 7 Department of Optometry and Visual Science, City University, Northampton Square, London EC1V 0HB, United Kingdom

**Keywords:** HUMDISEASE

## Abstract

As the ear has dual functions for audition and balance, the eye has a dual role in detecting light for a wide range of behavioral and physiological functions separate from sight [1–11]. These responses are driven primarily by stimulation of photosensitive retinal ganglion cells (pRGCs) that are most sensitive to short-wavelength (∼480 nm) blue light and remain functional in the absence of rods and cones [8–10]. We examined the spectral sensitivity of non-image-forming responses in two profoundly blind subjects lacking functional rods and cones (one male, 56 yr old; one female, 87 yr old). In the male subject, we found that short-wavelength light preferentially suppressed melatonin, reset the circadian pacemaker, and directly enhanced alertness compared to 555 nm exposure, which is the peak sensitivity of the photopic visual system. In an action spectrum for pupillary constriction, the female subject exhibited a peak spectral sensitivity (λ_max_) of 480 nm, matching that of the pRGCs but not that of the rods and cones. This subject was also able to correctly report a threshold short-wavelength stimulus (∼480 nm) but not other wavelengths. Collectively these data show that pRGCs contribute to both circadian physiology and rudimentary visual awareness in humans and challenge the assumption that rod- and cone-based photoreception mediate all “visual” responses to light.

## Results and Discussion

**Figure 1:**
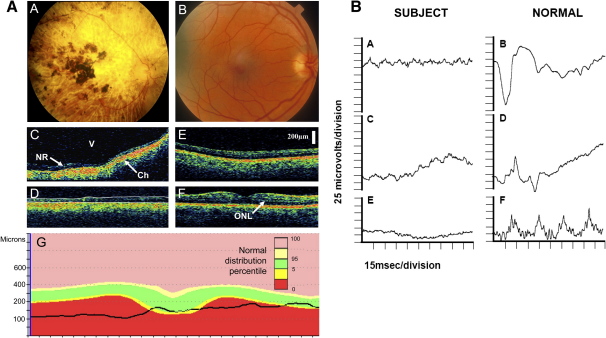
Neuroophthalmology and Ocular Anatomy of the Blind Female Subject and a Normal Control The left panel shows fundoscopy findings of the 87-year-old blind female subject (A) and a representative ocular-coherence tomogram for the peripheral retina (C) and central macula region (D) of the left eye, compared with a normal age-matched sighted control (B, E, and F). Her retina is abnormally thin (less than 160 microns) and there is no identifiable outer nuclear layer or photoreceptor layer, suggesting that photoreceptors are absent, and the choroid has abnormally high reflectivity (Ch) in contrast to the normal age-matched subject (E and F), where stratification within the neurosensory retina, particularly the outer nuclear layer (ONL), can be seen. By contrast, the ganglion cell and nerve fiber layers of the inner retina of the blind woman are of normal thickness, and there is no cellular disruption, allowing clear recognition and delineation of normal histo-architecture in both retinal periphery and macula. In (G), comparison of the normal macula profile in an age-matched individual (within green limits, as shown in OCT image in [F]) illustrates loss of normal macular contour in the blind subject (black line, as derived from [D]). The normal distribution percentile correlates the color-coded areas of the figure to percentages of age-matched people who might possess retinae within that region. V = vitreous, NR = neurosensory retina. The right panel shows electroretinographic responses from the female subject (A, C, and E) and an age-matched, normal eye (B, D, and F) for dark-adapted (rod-photoreceptor predominant) responses (A and B); dark-adapted, light-adapted (mixed photoreceptor) responses (C and D); and light-adapted (cone predominant) responses (E and F) to 30 Hz flicker stimuli. White-light stimuli at 3.0 cd s/m^2^ intensity were used for all tests and began at the start of recordings in all cases. The traces for the blind subject show no detectable electroretinographic responses (Note: [C]shows a drifting baseline.).

Two blind subjects (one male, 56 yr old; one female, 87 yr old) without light perception were studied in parallel experiments. The female subject was a member of a family expressing an autosomal-dominant cone-rod dystrophy, which is described as a severe, early-onset phenotype with patients progressing to no perception of light by the fifth decade of life [12, 13]. The male subject had retinitis pigmentosa, a progressive disease of the retinal photoreceptors, and he reported losing light perception in his mid-30s. He had bilateral posterior subcapsular cataracts. Both subjects met all clinical criteria of blindness arising from degenerative retinal disease. These include pupils that are unreactive to light after standard penlight examination and self-reported inability to perceive light. Fundus photography and ocular coherence tomography failed to identify an outer retina in the female subject (an absence consistent with blindness), and electroretinography demonstrated no detectable rod or cone function (Figure 1). A fundoscopic examination of the male subject also revealed atrophy of the retinal pigment epithelium layer throughout the fundi, and visually evoked potentials were negative, again consistent with total visual loss.

**Figure 2:**
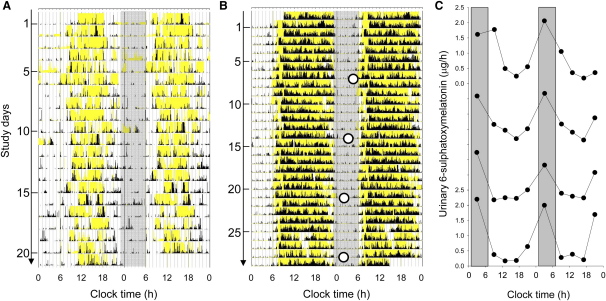
Entrained Rest-Activity and Urinary 6-Sulphatoxymelatonin Rhythms in Two Blind Subjects The daily activity rhythm (black) and light (lux) exposure (yellow) patterns of the female (A) and male (B) subjects, recorded at home for 3–4 weeks with wrist actigraphy (Actiwatch-L, Minimitter, New York). Data are double-plotted, with consecutive days plotted next to and beneath each other. The gray bars represent an arbitrary “night” from 23:00–6:00 hr for visual reference. Analysis of actigraphy data indicated that both the female and male subject had sleep onset (mean ± standard deviation [SD] sleep onset = 21:50 ± 1:09 hr and 23:22 ± 0:24 hr, respectively) and sleep offset (8:38 ± 1:29 hr and 6:31 ± 0:26 hr, respectively) times that fell within the range of actigraphically derived sleep times for blind subjects with previously confirmed normally phased circadian sleep and urinary 6-sulphatoxymelatonin rhythms (mean ± 2SD sleep onset = 23:31 ± 2:26 hr, sleep offset = 7:11 ± 2:24 hr) [3, 14]. The urinary 6-sulphatoxymelatonin (aMT6s) rhythm peak time (○) in the male subject confirmed the presence of a normally phased nighttime 24 hr rhythm (mean ± SD = 3:00 ± 1:17 hr) that exhibited a normal phase angle (3:38 hr) with respect to the sleep/wake cycle based on previous studies in entrained blind subjects (mean ±2SD phase angle, sleep onset − aMT6s peak = 4:38 ± 2:28 [3, 14]). The raw urinary data are shown in [C] with the normal peak-time range for the aMT6s rhythms shown in gray (1:42–6:36 hr) [3].

Both subjects reported having no sleep disorders and normal age-appropriate 24-hr sleep/wake patterns, as confirmed by quantitative assessments of circadian rest-activity behavior carried out with wrist actigraphy while they lived at home [14, 15]; these results are consistent with a functionally intact retinohypothalmic tract [1, 16] (Figure 2). A normal circadian phase was further confirmed using urinary 6-sulphatoxymelatonin (aMT6s) rhythms in the male subject [3] (Figure 2; also, Supplemental Data available online).

**Figure 3:**
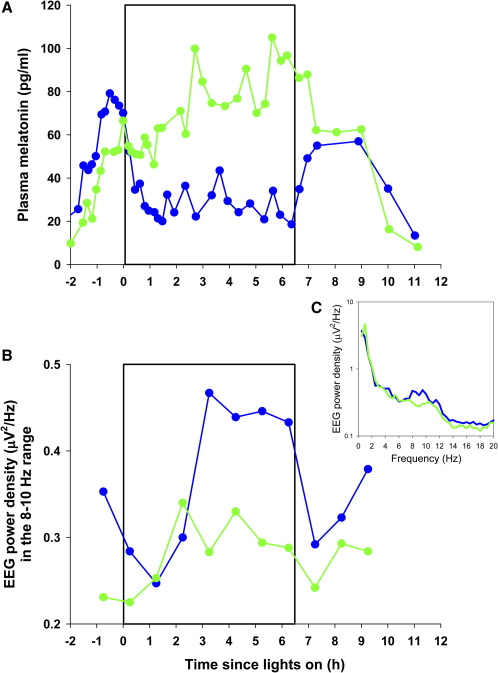
Short-Wavelength Light Sensitivity for Melatonin Suppression and Enhancement of EEG Alpha Power in a Blind Man The direct effects of exposure to green (555 nm) and blue (460 nm) monochromatic light on the male subject for melatonin suppression (A) and waking-EEG power density (B) as an objective correlate of alertness. Exposure to 555 nm light caused no suppression of melatonin as compared to the corresponding clock time the previous day, whereas exposure to 460 nm light suppressed melatonin (total suppression by AUC = 57%) and maintained the suppression effect throughout the entire 6.5-hr exposure (A). The 460 nm light also caused an elevation of alpha activity (8–10 Hz) in the waking EEG, indicative of a more alert state (B). Only alpha frequencies exhibited a wavelength-dependent difference during the second half of the light exposure (C). These data are consistent with the short-wavelength sensitivity for the acute effects of light in sighted subjects under similar conditions [17, 18, 21].

In experiment 1, conducted with the male subject, we aimed to test the spectral sensitivity of the circadian, neuroendocrine, and neurobehavioral axes (Figure 3 and Figure S1). First, we confirmed that he retained a normal melatonin-suppression response to bright-white light exposure [1] on two separate occasions three years apart (see Supplemental Data). We then conducted a 14 day inpatient study to compare the effects of 6.5 hr exposure to 460 nm and 555 nm monochromatic light on circadian phase resetting, melatonin suppression, and enhancement of arousal [17, 18]. In order to compare the relative contribution of the photosensitive retinal ganglion cells (pRGCs) and classical (rod/cone) photoreceptors, we chose two light sources that would differentially stimulate these systems: a monochromatic “blue” light source with a peak emission (λ_max_) at 460 nm and hence close to the λ_max_ of human pRGCs (∼480 nm) [11, 19], and a monochromatic light source with a λ_max_ at 555 nm corresponding to the peak of human photopic vision. Given that this subject exhibited a 24-hr sleep-wake pattern and an entrained aMT6s rhythm, we predicted that the pRGC/melanopsin-driven system would be intact and that the short-wavelength stimulus would elicit full circadian, neuroendocrine, and neurobehavioral responses, whereas the lack of classical photoreception would preclude any response to mid-wavelength 555 nm light.

In a randomized, single-blind design, we exposed the subject to an equal photon density (2.8 × 10^13^ photons/cm^2^/s) of 555 nm and 460 nm monochromatic light for 6.5 hr, timed to start 1.25 hr before the prestudy bedtime [17, 18]. The subject was seated 90 min prior to and during light exposure, and for 60 min afterward, and was administered a pupil dilator (1 drop per eye, 0.5% cyclopentolate HCl; Cyclogyl, Alcon Laboratories, Texas) and kept in darkness for 15 min prior to lights on (see Supplemental Data). As hypothesized because of the absence of a functional cone response, ocular exposure to 555 nm light had no effect on plasma melatonin, whereas 460 nm light suppressed melatonin by 57% (Figure 3A). Exposure to 460 nm light also caused a −1.2 hr phase delay in the timing of the circadian melatonin rhythm, whereas 555 nm light caused a minimal phase shift (−0.4 hr). In addition, the blue light preferentially increased alpha activity (8–10 Hz) in the waking electroencephalogram (EEG) recordings, indicating a more alert state [18, 20] (Figure 3B), and appeared to decrease subjective sleepiness and improve auditory performance during the latter half of the light exposure (Figure S1), consistent with the short-wavelength sensitivity for the acute effects of light in sighted subjects under similar conditions [17, 18, 21]. It is interesting to note that the blue light did not cause a suppression of delta and theta activity in the waking EEG, as we have previously observed in sighted subjects [18], and it is tempting to suggest that the lack of rod-cone photoreception in this subject may account for the altered EEG response at those particular frequencies, as we recently speculated [22]. Further data are required, however, to confirm this hypothesis. Nevertheless, the short-wavelength near-maximal sensitivity to light at this photon density for a range of responses indicates that this blind subject has a fully functional non-rod, non-cone photoreceptor system mediating the circadian, neuroendocrine, and neurobehavioral effects of light, presumably via intact melanopsin-containing pRGCs.

**Figure 4:**
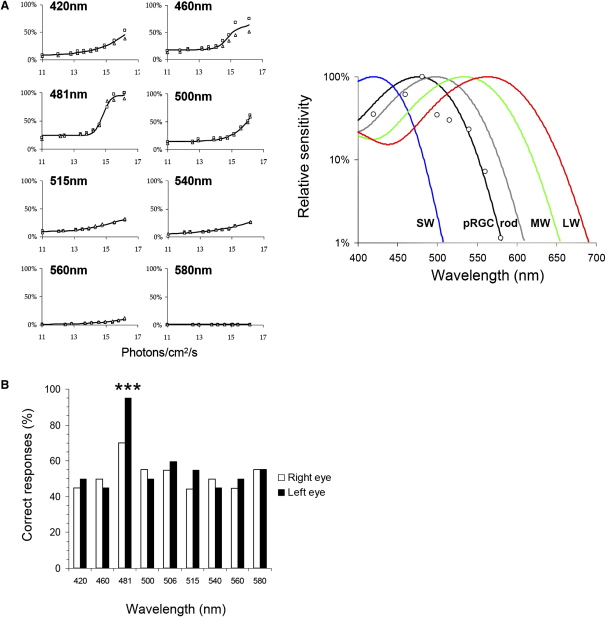
Short-Wavelength Light Sensitivity for Pupillary Constriction and Light Detection in a Blind Woman Irradiance-response curves (IRCs) were conducted at eight wavelengths for both eyes (squares indicate left eye, triangles indicate right eye) (A, left panel). Responses are plotted as percentage of maximum response obtained. IRCs were fitted with a four-parameter sigmoid function, with R^2^ values >0.90 in all cases. The resulting action spectrum of pupil responses (A, right panel) provided a poor fit to rod and cone photopigments (rod R^2^ = 0.35; SW cone, MW cone, LW cone R^2^ = 0). An optimum fit to the pupil response to light was provided by an opsin/vitamin A-based template with λ_max_ 476 nm (R^2^ = 0.89), corresponding closely to the pRGC system. Note: Data shown were not corrected for preretinal lens absorption. When this correction was applied, the λ_max_ shifted from 476 nm to 480 nm. (B) shows the results of the psychophysical testing in the same subject that indicated conscious perception of light at 481 nm (^∗∗∗^p < 0.001) but failure (p > 0.05) to detect light at longer or shorter wavelengths (420, 460, 500, 515, 540, 560, and 580 nm). These results mirror the spectrally tuned response of the pupil, and suggest that the subject's detection and awareness of light also arise from pRGCs. Each histogram represents the percentage of correct responses out of 20 trials for both left and right eyes (360 trials in total).

In experiment 2, we investigated the spectral sensitivity of pupil construction in the female subject by using analytical-photobiological action-spectroscopy techniques. On the basis of her 24 hr sleep/wake pattern and our previous studies on rodents [9, 23], we reasoned that she should also possess some pupil reactivity to bright light, despite the clinical reports that she was unresponsive to the brief light exposure from either a penlight or indirect ophthalmoscopic examination. Quantitative pupillometry, employing monochromatic light at a broad range of wavelengths and irradiances (10^11^–10^16^ log photons/cm^2^/s) with an exposure duration of 10 s, showed that the subject possessed a functioning pupillomotor system responsive to bright light. The pupil-constriction response was spectrally tuned, peaking (λ_max_) at 476 nm. Irradiance-response curves showed a high statistical fit of their derived half-saturation constants to a vitamin A opsin-pigment nomogram (R^2^ = 0.89, compared to R^2^ = 0.35 for rod and R^2^ < 0.01 for all three cone classes), suggesting that pupil constriction was being driven by a single photopigment (Figure 4). The spectral maxima of 476 nm corresponds well to the action spectra for pRGCs in both human (483 nm) and nonhuman primates (482 nm) [10, 24], but not the λ_max_ of human rods (∼498 nm) or short, medium, and long-wavelength cones (λ_max_ ∼420, 534, and 563 nm, respectively) [25] (Figure 4). When the pupil-action spectrum was corrected for preretinal lens absorption [26], the peak spectral sensitivity shifted slightly from 476 nm to 480 nm. Consistent with the results from experiment 1, these data show that this subject possesses both an intact retinopretectal projection (pupillary constriction) and a retinohypothalamic projection (circadian entrainment), and that these responses to light are driven exclusively by short-wavelength-sensitive pRGCs in subjects lacking rods and cones and do not require input from the photopic system [24]. Notably, the confirmation of a pupil response following longer-duration exposure than typically used in brief penlight examinations questions the relevance of this technique, given that unreactive pupils are considered clinically to be a sine qua non of profound blindness of retinal origin despite earlier evidence for short-wavelength sensitivity in human pupil responses [27, 28].

The recent finding in primates that the pRGCs project to the dorsal lateral geniculate nucleus (dLGN) [10]—the thalamic relay that provides a direct input to the visual cortex—led us to explore the possibility that these photoreceptors might contribute to an individual's ability to detect or even experience some awareness of light. We therefore tested whether the female subject could report whether a given light stimulus was present in the first or second of two temporal intervals in a two-alternative forced-choice paradigm (2AFC). After some initial hesitancy about being asked to report the presence of visual stimuli of which she was nominally unaware, she was able to correctly identify the interval in which a 481 nm test light appeared (p < 0.001) but failed (p > 0.05) to detect light at longer or shorter wavelengths (420, 460, 500, 515, 540, 560, and 580 nm) (Figure 4). These detection probabilities remained unchanged when corrected for multiple testing (Bonferroni). Furthermore, she reported that the presence of the detectable stimuli (481 nm) elicited in her a percept that she described as “brightness.” Although superficially these responses resemble cortical blindsight in that she was able to detect a stimulus with a rate of success above chance [29], these data represent a markedly different phenomenon because subjects with damage to the primary visual cortex (V1) have no conscious perception of the stimulus presented [29].

Could these responses to light have arisen from a small number of surviving rods and/or cones rather than from the pRGCs? Although visually evoked potentials (VEP), electroretinogram (ERG), and ocular coherence tomography (OCT) analysis cannot preclude the persistence of a residual population of rods and/or cones, there was no functional evidence of any significant rod or cone involvement. Both the λ_max_ of ∼480 nm and the correspondence of the action spectrum to a single opsin- and vitamin A-based photopigment template strongly implicate phototransduction by the pRGC subsystem alone.

The question remains, however, which neuronal pathways and brain structures mediate these “nonvisual” effects of light. Neuroanatomical investigations in rodents show that melanopsin-containing ganglion cells project to a range of retinorecipient nuclei, including major projections to (1) the hypothalamic suprachiasmatic nuclei (SCN), the site of endogenous circadian pacemaker; (2) the intergeniculate leaflet of the thalamus, an area that is closely linked to normal circadian function and conveys photic and nonphotic signals to the SCN; (3) the ventrolateral preoptic area, an area that controls the switch between sleep and wake states; (4) the olivary-pretectal nucleus, implicated in the pupillary constriction response; and (5) the superior colliculus, which mediates visual and auditory sensorimotor reponses [30, 31]. As indicated previously, a subset of melanopsin-containing ganglion cells also project to the dLGN [10, 31] and in primates have a peak spectral sensitivity (λ_max_) of 482 nm [10], thereby possibly providing the neuroanatomical substrate in support of the identical short-wavelength sensitivity for the visual awareness response observed in the female subject. Moreover, recent imaging studies in humans are beginning to identify brain regions associated with light-induced improvements in performance and cognition [32–34] and show preferential short-wavelength activation of the thalamus and the anterior insula, structures strongly implicated in arousal and memory function [34].

Our data strengthen the conclusion that the clinical diagnosis of ”complete” blindness (i.e., visual and circadian) should assess the state of both the image-forming and the non-image-forming photoreceptive systems [1]. If blind individuals are found to be light sensitive, this knowledge will help ensure that they expose their eyes to sufficient daytime light to maintain normal circadian entrainment and sleep/wake rhythmicity. This evaluation is particularly critical prior to bilateral enucleation because, if light-responsive eyes are removed or individuals do not expose their eyes to a robust light-dark cycle, the patients may develop a debilitating circadian-rhythm sleep disorder [3, 14]. Patients with diseases of the inner retina that result in retinal ganglion cell death (e.g., glaucoma) are at particular risk and should be counseled about the effects of pRGC loss. Where complete blindness results, appropriately timed melatonin treatment may be warranted in order to establish entrained circadian rhythmicity [35, 36].

## Conclusions

We have shown that circadian, neuroendocrine, and neurobehavioral responses to light, and even visual awareness of light, are retained in visually blind subjects lacking functional outer retinae, confirming in humans the recent remarkable discovery of a novel photoreceptor system in the mammalian eye. These findings question the traditional view that rod- and cone-based photoreception mediate all “visual” responses to light (such as pupillary constriction and visual awareness) and suggest that these and “nonvisual” circadian and neuroendocrine responses to light in humans are driven primarily by a non-rod, non-cone, short-wavelength-sensitive photoreceptor system located in the ganglion cell layer.

## Supplemental Data

Document S1. Experimental Procedures and One Figure
