# Supplementary material for: Short-Wavelength Light Sensitivity of Circadian, Pupillary, and Visual Awareness in Humans Lacking an Outer Retina
Source: Curr Biol. 2007 Dec 18;17(24):2122–8. doi: 10.1016/j.cub.2007.11.034 (PMC2151130; doi:10.1016/j.cub.2007.11.034)
Supplement: Document S1. Experimental Procedures and One Figure [file mmc1.pdf]

Supplemental Data

Short-Wavelength Light Sensitivity of Circadian, Pupillary, and Visual Awareness in Humans Lacking an Outer Retina

Farhan H. Zaidi, Joseph T. Hull, Stuart N. Peirson, Katharina Wulff, Daniel Aeschbach, Joshua J. Gooley, George C. Brainard, Kevin Gregory-Evans, Joseph F. Rizzo III, Charles A. Czeisler, Russell G. Foster, Merrick J. Moseley, and Steven W. Lockley

Supplemental Experimental Procedures

Ophthalmological Examinations

Ocular coherence tomography (OCT) was conducted in the female subject with a third-generation interferometric, noninvasive optical tomographic imaging device (OCT3 Zeiss Humphrey Division, Dublin, California) providing 2–3 mm tissue penetration. Axial and lateral resolution was < 10–20 microns. Electroretinographic responses were recorded to International Society for Clinical Electrophysiology of Vision (ISCEV) standards [S1] with RETI-port basic electrophysiological diagnostic systems (Roland Instruments, Brandenburg, Germany) and DTL (Dawson Trick Litzkow) fiber corneal electrodes. Visually evoked potentials (VEPs) were assessed in the male subject via both pattern reversal (2/s) and strobe (2/s) with a high-contrast black-white checkerboard. EEG recordings were made from Fz-Oz, Pz-Oz, Fz-O1, and Fz-O2. No P100 responses were obtained, indicating no light perception in either eye.

Experiment 1a: White-Light Melatonin-Suppression Test

Two white-light melatonin-suppression tests were conducted three years apart during two different inpatient studies in the Intensive Physiology Monitoring (IPM) unit of the General Clinical Research Center (GCRC) at Brigham and Women's Hospital. The subject (22CS) was exposed to 10,000 lux white fluorescent light (4100K, Philips Lighting, The Netherlands) for 6.5 hr during the biological night (21:75–04:25 hr), centered in a 16 hr wake episode in dim light (~4 lux). The subject was seated 90 min prior to and 60 min after the light exposure and was asked to fix his "gaze" toward the light for 50% of the exposure duration in an alternating "fixed:free" gaze pattern. During the free gaze, he was encouraged to avoid photophobic behavior. Light measurements were made at the level of the eye with an IL1400 radiometer/powermeter with an SEL-033/Y/W detector (International Light, Massachusetts). Plasma melatonin was sampled every 20–60 min via an indwelling catheter kept patent with a heparinized saline drip (5 IU heparin/mL 0.45% NaCl). Melatonin suppression was calculated by dividing the percent difference between the area under the curve (AUC) of the plasma-melatonin profile during the 6.5 hr light exposure by the AUC during the same clock time while the subject was awake under constant routine conditions in dim light (~4 lux) the previous day.

Exposure to 10,000 lux white light for 6.5 hr caused a 90% and 86% reduction in plasma-melatonin levels, respectively, consistent with the normal response in fully sighted subjects studied under similar conditions [S2].

Experiment 1b: Circadian, Neuroendocrine, and Neurobehavioral Response to Monochromatic Light Exposure

The effects of monochromatic light exposure were studied during a 14 day inpatient protocol in an environment free of time cues (individual windowless, soundproofed suite) in the GCRC IPM at Brigham and Women's Hospital. The subject was healthy except for his visual impairment, as determined from physical and psychological examinations as well as blood and urine toxicology. For 4 weeks prior to admission, he maintained a consistent 8 hr sleep episode, as confirmed with wrist actigraphy (Actiwatch-L, Minimitter, New York) and sleep-wake logs, and refrained from any drug use, including caffeine, alcohol, nicotine, dietary supplements, and prescription or nonprescription medications, as confirmed by admission toxicology. The 14 day inpatient study consisted of two consecutive identical 7 day schedules with the experimental light exposures occurring 6 days apart. Given the potential for adaptation of the circadian photoreception system by prior light history [S3–S6], the protocol was designed to ensure that light conditions were identical before each experimental light exposure.

For the first 3 days, the subject was scheduled for 8 hr sleep episodes in darkness (timed according to the prestudy sleep schedule) and 16 hr wake episodes in ~90 lux white light (measured at a height of 137 cm in the horizontal angle of gaze). Eight hours after waking on day 3, ambient light levels were reduced to 4 lux. Upon waking on day 4, the subject began a 26 hr constant routine (CR) procedure [S7] to measure the endogenous circadian rhythm of melatonin. During the CR procedure, the subject was continually supervised while remaining semirecumbant and awake in bed under dim light (~4 lux) and provided identical hourly snacks in order to abolish or distribute equally environmental circadian time cues. After an 8 hr sleep episode, the subject began a 16 hr wake episode in dim light, except during the 6.5 hr monochromatic light exposure centered in the wake episode. Following an 8 hr sleep, the subject completed a second CR procedure for 30 hr before an 8 hr sleep at his habitual bedtime. This 7 day sequence was then repeated under identical conditions.

The experimental light-exposure conditions were identical for the white light studies except that the subject was exposed to monochromatic light continuously for 6.5 hr via a modified Ganzfeld source [S8, S9], 15 min after administration of a mydriatic. The order of the monochromatic light exposure (555 nm, 460 nm) was randomly determined but not revealed to the subject. Light wavelength and irradiance were measured with a PR-650 SpectraScan Colorimeter with a CR-650 cosine receptor (PhotoResearch, California) and an IL1400 radiometer/powermeter with an SEL-033/F/W detector (International Light, MA), respectively. Plasma melatonin was drawn every 20–60 min, subjective sleepiness was assessed every 30 min with the Karolinksa Sleepiness Scale, and auditory performance was measured with a 10 min Psychomotor Vigilance Test (PVT) every hour while the subject was awake. Waking-EEG recordings were conducted during most wake episodes and included a 3 min Karolinska Drowsiness Test each hour with a portable digital polysomnographic recorder (Vitaport-3 digital recorder, TEMEC Instruments B.V., The Netherlands). Recordings consisted of EEG, electrooculogram, and a 2-lead electrocardiogram (ECG), and electrodes were positioned according to the International 10-20 System, with linked mastoid references (Ax) from the z-line, Fz-Ax, Cz-Ax, Pz-Ax, and Oz-Ax. Power-density spectra were calculated for 2 s epochs with a Fast Fourier Transform routine (Vitagraph, TEMEC, The Netherlands) and averaged within the 3 min KDT intervals, after visually identified artifacts were excluded. The data presented were re-referenced offline using the Cz-Pz derivation because of ECG artifact in the Ax recordings. For further details, see [S10].

Plasma melatonin rhythms during the inpatient baseline days and initial circadian phase assessment confirmed the normal circadian entrainment indicated by the home-based urinary 6-sulpatoxymelatonin rhythms (Figure 2 in the main text), with an average dim-light melatonin onset (DLMO) time of 20.70 ± 0.08 hr (n = 3 days). The circadian phase of the two experimental light exposures was also consistent, as confirmed by the DLMO phase assessments prior to each light exposure (20.28 hr and 19.78 hr, respectively).

Experiment 2a: Pupillary-Constriction Action Spectroscopy

Direct, monocular constriction responses to light stimulation were recorded at ten irradiance intensities (10^{11}–10^{16} log photons/cm^2/s) for each of eight wavelengths (420, 460, 481, 500, 515, 540, 560, and 580 nm). Pupil area was monitored with a PSCAN_100 (Circuit

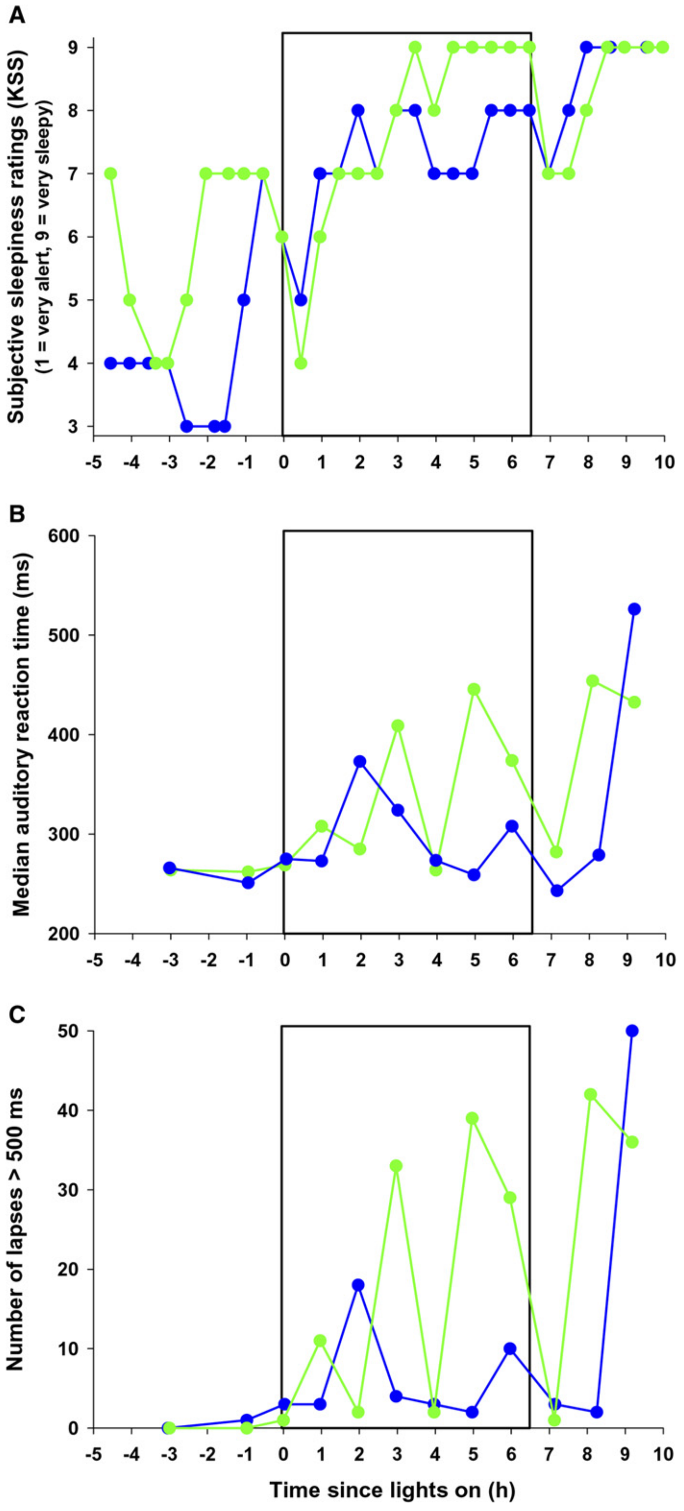

Figure S1. Short-Wavelength Sensitivity for the Acute Effects of Light on Sleepiness and Auditory Performance in a Blind Man

The direct effects of exposure to green (555 nm) and blue (460 nm) monochromatic light on the male subject for subjective sleepiness (A), auditory reaction time (B), and lapses of attention (C) plotted according to the same convention as that for melatonin suppression in Figure 3 (main text). Subjective sleepiness appeared to decrease during the latter half of the 460 nm exposure, as compared to both the previous day and directly with 555 nm exposure (A). Similarly, auditory median reaction time (B) and lapses of attention (C) also showed greater improvement during 460 nm light exposure over the second half of the exposure, as compared to 555 nm light exposure, consistent with the changes in EEG alpha activity at the same time (Figure 3 in the main text). These data are consistent with the short-wavelength sensitivity for the acute effects of light in sighted subjects under similar conditions.

Solve, UK) infrared pupillometer with a spatial resolution of 0.05 mm. Stimuli were of 10 s duration and provided by a quartz halogen light source delivered in non-Maxwellian view. Irradiance and wavelength were controlled with neutral density and monochromatic interference filters, respectively (10 nm half-peak bandwidth).

Irradiance-response curves (IRCs) were generated by plotting pupil constriction (y axis) against light dose (log photons/cm^2/s). IRCs were fitted with a four-parameter sigmoid curve: y = a + (b/[1+10(c-x)/d]), where y is the response, a is the baseline, b is the maximum response, c is the irradiance required to evoke a 50% pupil constriction (IR_{50}), x is the photon flux (log photons/cm^2/s) for which y is to be derived, and d is the slope of the curve. Each curve was fitted in MS Excel (Microsoft), with Solver add-in (Frontline systems) used to minimize the sum of squares for each IRC, as described previously [S11]. R^2 values were > 0.90 for all IRCs (Figure 4A in the main text). An action spectrum was then constructed on the basis of relative sensitivities derived from IRCs at the eight wavelengths investigated (Figure 4A in the main text). Given that responses reached half-maximum at only three wavelengths, relative sensitivity based upon IR_{50} would result in

extrapolation beyond the data range. Consequently, relative sensitivity was determined from responses obtained from an equivalent photon flux in the dynamic range of the IRC (i.e., above background noise and before saturation), as described previously [S12]. Data were analyzed on the basis of responses to 1 x 10^{15} photons/cm^2/s, although comparable results were obtained within the range of 1 × 10^{14}–1 × 10^{16} photons/cm^2/s.

Absorbance spectra for human visual pigments were generated using the Govardovskii template [S13], based upon the published λ_{max} values [S14] of rod (498 nm) and of short- (420 nm), mid- (534 nm), and long-wavelength cone (563 nm). Free-fitting of the visual pigment template was conducted via the least-squares method described above, with log-transformed sensitivity and template data used to prevent peak-fitting bias. Given that preretinal absorption measurements were not available for the subject, the data shown were not corrected for preretinal lens absorption. When standardized corrections were applied [S15], the λ_{max} shifted from 476 nm to 480 nm, although the R^2 decreased to 0.70 as a result of the differential filtering effects at 420 nm.

Experiment 2b: Forced-Choice Paradigm for Light Detection

The subject's ability to detect and/or perceive light stimuli of different wavelengths was determined by a two-alternative forced-choice (2AFC) procedure. In this procedure, a light stimulus is presented in one of two temporal intervals, and it is mandatory for the subject to state at which interval he or she believes the stimulus to have been presented. Stimuli lasted for 10 s at an intensity of 1.45 × 10^{16} photons/cm^2/s at all wavelengths. Twenty trials were conducted for each of nine wavelengths for each eye. Data were analyzed with the Sign test, with a probability of level of 0.05 (corresponding to correctly identifying the stimulus interval in 15/20 trials) deemed to be indicative of statistically significant detection. Note: These methods were relatively lengthy and taxing on this elderly subject. We therefore selected a photon flux, based upon pilot experiments on the subject, that produced a significant but not saturating discriminatory response. Responses to the 2AFC procedure are not graded, and there is a tendency with this experimental paradigm to exaggerate above-threshold stimuli and ignore near-threshold stimuli. The results obtained reflect this tendency. The approach allowed us to determine the wavelength of light that would allow a threshold response rather than a complete response profile.

References

S1. Marmor, M.F., Holder, G.E., Seeliger, M.W., and Yamamoto, S. (2004). Standard for clinical electroretinography (2004 update). Doc. Ophthalmol. *108*, 107–114.

S2. Zeitzer, J.M., Dijk, D.J., Kronauer, R., Brown, E., and Czeisler, C. (2000). Sensitivity of the human circadian pacemaker to nocturnal light: Melatonin phase resetting and suppression. J. Physiol. *526*, 695–702.

S3. Hankins, M.W., and Lucas, R.J. (2002). The primary visual pathway in humans is regulated according to long-term light exposure through the action of a nonclassical photopigment. Curr. Biol. *12*, 191–198.

S4. Smith, K.A., Schoen, M.W., and Czeisler, C.A. (2004). Adaptation of human pineal melatonin suppression by recent photic history. J. Clin. Endocrinol. Metab. *89*, 3610–3614.

S5. Wong, K.Y., Dunn, F.A., and Berson, D.M. (2005). Photoreceptor adaptation in intrinsically photosensitive retinal ganglion cells. Neuron *48*, 1001–1010.

S6. Jasser, S.A., Hanifin, J.P., Rollag, M.D., and Brainard, G.C. (2006). Dim light adaptation attenuates acute melatonin suppression in humans. J. Biol. Rhythms *21*, 394–404.

S7. Duffy, J.F., and Dijk, D.J. (2002). Getting through to circadian oscillators: Why use constant routines? J. Biol. Rhythms *17*, 4–13.

S8. Brainard, G.C., Hanifin, J.P., Greeson, J.M., Byrne, B., Glickman, G., Gerner, E., and Rollag, M.D. (2001). Action spectrum for melatonin regulation in humans: Evidence for a novel circadian photoreceptor. J. Neurosci. *21*, 6405–6412.

S9. Lockley, S.W., Brainard, G.C., and Czeisler, C.A. (2003). High sensitivity of the human circadian melatonin rhythm to resetting by short wavelength light. J. Clin. Endocrinol. Metab. *88*, 4502–4505.

S10. Lockley, S.W., Evans, E.E., Scheer, F.A., Brainard, G.C., Czeisler, C.A., and Aeschbach, D. (2006). Short-wavelength sensitivity for the direct effects of light on alertness, vigilance, and the waking electroencephalogram in humans. Sleep *29*, 161–168.

S11. Peirson, S.N., Thompson, S., Hankins, M.W., and Foster, R.G. (2005). Mammalian photoentrainment: results, methods, and approaches. Methods Enzymol. *393*, 697–726.

S12. Provencio, I., and Foster, R.G. (1995). Circadian rhythms in mice can be regulated by photoreceptors with cone-like characteristics. Brain Res. *694*, 183–190.

S13. Govardovskii, V.I., Fyhrquist, N., Reuter, T., Kuzmin, D.G., and Donner, K. (2000). In search of the visual pigment template. Vis. Neurosci. *17*, 509–528.

S14. Bowmaker, J.K., and Dartnall, H.J. (1980). Visual pigments of rods and cones in a human retina. J. Physiol. *298*, 501–511.

S15. Stockman, A., Sharpe, L.T., and Fach, C.C. (1999). The spectral sensitivity of the human short-wavelength cones. Vision Res. *39*, 2901–2927.
